# *N*-(4-{4-[2-(Trifluoro­meth­oxy)phen­yl]piperazin-1-yl}but­yl)thio­phene-2-carboxamide dihydrate

**DOI:** 10.1107/S160053681005155X

**Published:** 2010-12-15

**Authors:** Jin Cai

**Affiliations:** a School of Chemistry and Chemical Engineering, Institute of Pharmaceutical Engineering, Southeast University, Nanjing 210096, People’s Republic of China

## Abstract

In the title compound, C_20_H_24_F_3_N_3_O_2_S·2H_2_O,  a dopamine D3 ligand, the piperazine ring adopts a chair conformation while the piperazine and benzene rings form a dihedral angle of 47.71 (6)°. In the crystal, mol­ecules are linked by inter­molecular N—H⋯O and O—H⋯O hydrogen bonds. In the mol­ecular structure, the F atoms of the trifluoro­methyl group are disordered over two sites with occupancies of 0.69 (11) and 0.31 (11).

## Related literature

For the synthesis of the title compound and its derivatives, see: Leopoldo *et al.* (2002 ▶); Robarge *et al.* (2001 ▶). For the pharmacological activity of the dopamine D3 ligand, see: Pilla *et al.* (1999 ▶); Garcia-Ladona & Cox (2003 ▶); Wood *et al.* (2000 ▶); Luedtke & Mach (2003 ▶). For structure–activity relationships for the dopamine D3 receptor, see: Bettinetti *et al.* (2002) ▶; Leopoldo *et al.* (2002 ▶); Dutta *et al.*, (2004 ▶). 
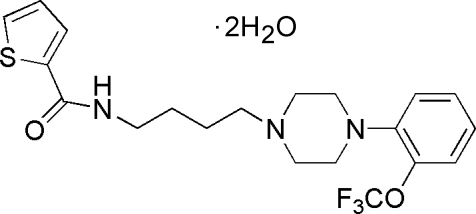


## Experimental

### 

#### Crystal data

C_20_H_24_F_3_N_3_O_2_S·2H_2_O
*M* *_r_* = 463.51
Orthorhombic, 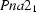

*a* = 9.361 (2) Å
*b* = 35.966 (9) Å
*c* = 6.9102 (17) Å
*V* = 2326.5 (10) Å^3^
*Z* = 4
Mo *K*α radiation
μ = 0.19 mm^−1^
*T* = 298 K
0.53 × 0.49 × 0.47 mm

#### Data collection

Bruker SMART CCD area-detector diffractometer
Absorption correction: multi-scan (*SADABS*; Bruker, 1999 ▶) *T*_min_ = 0.905, *T*_max_ = 0.915
11753 measured reflections
2234 independent reflections
1588 reflections with *I* > 2σ(*I*)
*R*_int_ = 0.051

#### Refinement

*R*[*F* ^2^ > 2σ(*F* ^2^)] = 0.056
*wR*(*F* ^2^) = 0.173
*S* = 1.05
2234 reflections
308 parameters
1 restraint
H-atom parameters constrained
Δρ_max_ = 0.28 e Å^−3^
Δρ_min_ = −0.34 e Å^−3^

### 

Data collection: *SMART* (Bruker, 1999 ▶); cell refinement: *SAINT* (Bruker, 1999 ▶); data reduction: *SAINT*; program(s) used to solve structure: *SHELXS97* (Sheldrick, 2008 ▶); program(s) used to refine structure: *SHELXL97* (Sheldrick, 2008 ▶); molecular graphics: *SHELXTL* (Sheldrick, 2008 ▶); software used to prepare material for publication: *SHELXTL*.

## Supplementary Material

Crystal structure: contains datablocks I, global. DOI: 10.1107/S160053681005155X/bg2379sup1.cif

Structure factors: contains datablocks I. DOI: 10.1107/S160053681005155X/bg2379Isup2.hkl

Additional supplementary materials:  crystallographic information; 3D view; checkCIF report

## Figures and Tables

**Table 1:** Hydrogen-bond geometry (Å, °)

*D*—H⋯*A*	*D*—H	H⋯*A*	*D*⋯*A*	*D*—H⋯*A*
N1—H1⋯O3^i^	0.86	2.17	2.929 (5)	148
O3—H21⋯N2^ii^	0.85	2.02	2.835 (4)	160
O3—H22⋯O1^iii^	0.85	1.98	2.822 (5)	171
O4—H23⋯O1^iv^	0.85	2.09	2.830 (7)	146
O4—H24⋯O3^v^	0.85	2.07	2.831 (6)	150

Symmetry codes: (i) 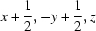; (ii) 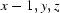; (iii) 
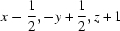
; (iv) 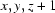; (v) 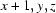.

**Fig. 1.**
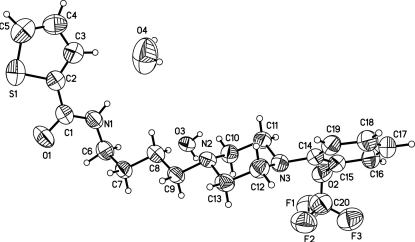
The molecular structure of the title compound, showing 30% probability displacement ellipsoids and the atom-numbering scheme. The minor part of the disordered moieties was omitted for clarity.

**Fig. 2.**
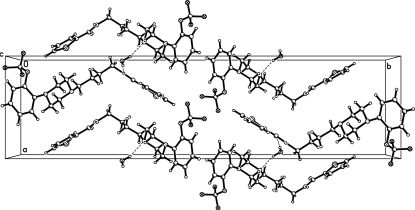
A packing diagram of the title compound. Intermolecular hydrogen bonds are shown as dashed lines. The minor part of the disordered moieties were omitted for clarity.

## supplementary crystallographic information

### Comment

The title compound was designed as a dopamine D3 ligand. The dopamine D3 receptor is recognized as a potential therapeutic target for the treatment of various neurological and psychiatric disorders, such as schizophrenia, Parkinson's disease and drug abuse (Pilla *et al.*, 1999; Garcia-Ladona *et al.*, 2003; Wood *et al.*, 2000; Luedtke *et al.*, 2003). Study of structure-activity relationships for dopamine D3 receptor are helpfull to rationalize the discovery of super-potent and highly selective dopamine D3 receptor antagonists and partial agonists (Bettinetti *et al.*, 2002; Leopoldo *et al.*, 2002; Dutta *et al.*, 2004). We report here the crystal structure of the title compound, which was synthesized by the two-component reaction of 4-(4-(2-(trifluoromethoxy) phenyl) piperazin-1-yl)butan-1-amine and thiophene-2-carbonyl chloride. In the title compound, the piperazine ring (C10/C11/C12/C13/N2/N3) adopts a chair conformation, atoms C10, C11, C12 and C13 are coplanar, with atoms N2 and N3 deviating from the plane by -0.661 (5) and 0.679 (5) Å, respectively (Fig. 1).The dihedral angle between the C10/C11/C12/C13 plane and the C15/C16/C17/C18/C19/C20 plane is 47.71 (6) °.The molecules are linked by N1–H1···O3 intermolecular hydrogen bonds (Table 1, Fig. 2). In the molecular structure, the fluorine atoms of trifluoromethyl group is disordered over two sites with occupancies of 0.69 (11) and 0.31 (11).

### Experimental

All chemicals used(reagent grade) were commercially available. 4-(4-(2-(trifluoromethoxy)phenyl)piperazin-1-yl)butan-1-amine 0.64 g (2mmol) was dissovled by 20 mL dichloromethane, then K_2_CO_3_ 2 g (15mmol) and thiophene-2-carbonyl chloride 0.29 g (2mmol) were added under ice-cooling and stirred for 30 min. The mixture was filtered, and evaporated the dissolvent.Purification of the crude product by a column chramotagraphy (*v:v* chloroform: methanol = 40:1) afforded the title compound (0.56g, 65.2%) Crystals suitable for X-ray diffraction were obtained by slow evaporation of an ethanol solution at room temperature. m.p. 358–359 K;^1^H-NMR(CDCl_3_, 300MHz), δ(ppm): 1.63-1.69(m, 4H), 2.47(t, 2H, *J*=6.93Hz), 2.61(t, 4H, *J*=4.74Hz), 3.09(t, 4H, *J*=4.82Hz), 3.47(q, 2H, *J*=6.26Hz), 6.36(br, 1H), 6.98-7.07(m, 3H), 7.18-7.26(m, 2H), 7.43-7.51(m, 2H); ^13^C-NMR(CDCl_3_, 300MHz), δ(ppm): 24.3, 27.6, 27.7, 40.0, 50.7, 50.8, 50.9, 53.4, 58.0, 119.9, 122.0, 122.3, 122.4, 122.5, 122.6, 127.5, 127.5, 127.9, 129.5, 139.2, 142.4, 145.1, 162.0; ESI-MS *m/z*: 428.2[M+H]^+^; Anal. calcd for C_20_H_24_F_3_N_3_O_2_S(%): C 56.19, H 5.66, N 9.83; Found: C 56.25, H 5.70, N 9.83.

### Refinement

All H atoms were placed in calculated positions, with O–H = 0.85 Å, N–H = 0.86 Å, and C–H = 0.93 or 0.97 Å, and included in the final cycles of refinement using a riding model, with U_iso_(H) = 1.2U_eq_(parent atom). The fluorine atoms of trifluoromethyl group is disordered over two sites with occupancies of 0.69 (11) and 0.31 (11). Due to the lack of any strong anomalous dispersor making absolute determination feasible, Friedel pairs were merged, thus leading to a rather poor data to parameters ratio.

### Crystal data

**Table:**

C_20_H_24_F_3_N_3_O_2_S·2H_2_O	*D*_x_ = 1.323 Mg m^−^^3^
*M**_r_* = 463.51	Melting point = 358–359 K
Orthorhombic, *P**n**a*2_1_	Mo *K*α radiation, λ = 0.71073 Å
Hall symbol: P 2c -2n	Cell parameters from 2897 reflections
*a* = 9.361 (2) Å	θ = 2.3–20.9°
*b* = 35.966 (9) Å	µ = 0.19 mm^−^^1^
*c* = 6.9102 (17) Å	*T* = 298 K
*V* = 2326.5 (10) Å^3^	Block, colorless
*Z* = 4	0.53 × 0.49 × 0.47 mm
*F*(000) = 976	

### Data collection

**Table:**

Bruker SMART CCD area-detector diffractometer	2234 independent reflections
Radiation source: fine-focus sealed tube	1588 reflections with *I* > 2σ(*I*)
graphite	*R*_int_ = 0.051
phi and ω scans	θ_max_ = 25.0°, θ_min_ = 2.3°
Absorption correction: multi-scan (*SADABS*; Bruker, 1999)	*h* = −11→10
*T*_min_ = 0.905, *T*_max_ = 0.915	*k* = −34→42
11753 measured reflections	*l* = −8→8

### Refinement

**Table:**

Refinement on *F*^2^	Primary atom site location: structure-invariant direct methods
Least-squares matrix: full	Secondary atom site location: difference Fourier map
*R*[*F*^2^ > 2σ(*F*^2^)] = 0.056	Hydrogen site location: inferred from neighbouring sites
*wR*(*F*^2^) = 0.173	H-atom parameters constrained
*S* = 1.05	*w* = 1/[σ^2^(*F*_o_^2^) + (0.0941*P*)^2^ + 1.1623*P*] where *P* = (*F*_o_^2^ + 2*F*_c_^2^)/3
2234 reflections	(Δ/σ)_max_ = 0.001
308 parameters	Δρ_max_ = 0.28 e Å^−^^3^
1 restraint	Δρ_min_ = −0.34 e Å^−^^3^

### Special details

**Table:**

Geometry. All esds (except the esd in the dihedral angle between two l.s. planes) are estimated using the full covariance matrix. The cell esds are taken into account individually in the estimation of esds in distances, angles and torsion angles; correlations between esds in cell parameters are only used when they are defined by crystal symmetry. An approximate (isotropic) treatment of cell esds is used for estimating esds involving l.s. planes.

### Fractional atomic coordinates and isotropic or equivalent isotropic displacement parameters (Å^2^)

**Table:**

	*x*	*y*	*z*	*U*_iso_*/*U*_eq_	Occ. (<1)
F1	0.466 (3)	0.4600 (11)	0.854 (8)	0.105 (9)	0.69 (14)
F2	0.674 (5)	0.4682 (14)	0.718 (5)	0.104 (8)	0.69 (14)
F3	0.612 (7)	0.5030 (6)	0.941 (4)	0.105 (7)	0.69 (14)
F1'	0.700 (8)	0.480 (3)	0.754 (16)	0.105 (16)	0.31 (14)
F2'	0.545 (14)	0.497 (2)	0.967 (8)	0.104 (18)	0.31 (14)
F3'	0.504 (10)	0.450 (2)	0.774 (16)	0.102 (19)	0.31 (14)
N1	0.6465 (5)	0.21413 (14)	0.2635 (8)	0.0622 (13)	
H1	0.6501	0.2112	0.3869	0.075*	
N2	0.8389 (5)	0.34518 (12)	0.5478 (7)	0.0494 (11)	
N3	0.8691 (5)	0.40422 (12)	0.8224 (7)	0.0513 (11)	
O1	0.7165 (5)	0.19256 (15)	−0.0274 (7)	0.0817 (14)	
O2	0.6543 (5)	0.44353 (11)	1.0101 (7)	0.0662 (12)	
O3	0.0358 (4)	0.28929 (11)	0.6596 (6)	0.0665 (11)	
H21	−0.0085	0.3096	0.6411	0.080*	
H22	0.0846	0.2933	0.7612	0.080*	
O4	0.9232 (9)	0.21670 (19)	0.7014 (14)	0.156 (3)	
H23	0.8935	0.2089	0.8104	0.187*	
H24	0.9764	0.2354	0.7235	0.187*	
S1	0.8756 (2)	0.12709 (6)	0.1079 (3)	0.0828 (6)	
C1	0.7140 (6)	0.18998 (17)	0.1526 (9)	0.0564 (15)	
C2	0.7881 (6)	0.15875 (17)	0.2491 (10)	0.0582 (16)	
C3	0.7965 (7)	0.14968 (18)	0.4386 (12)	0.0702 (19)	
H3	0.7577	0.1639	0.5379	0.084*	
C4	0.8706 (8)	0.1163 (2)	0.4675 (14)	0.080 (2)	
H4	0.8849	0.1058	0.5890	0.096*	
C5	0.9190 (8)	0.1009 (2)	0.3030 (15)	0.089 (3)	
H5	0.9698	0.0787	0.2971	0.107*	
C6	0.5661 (6)	0.24558 (17)	0.1865 (10)	0.0630 (16)	
H6A	0.4827	0.2498	0.2668	0.076*	
H6B	0.5333	0.2396	0.0571	0.076*	
C7	0.6549 (6)	0.28115 (16)	0.1791 (9)	0.0553 (14)	
H7A	0.7398	0.2766	0.1022	0.066*	
H7B	0.5998	0.3003	0.1146	0.066*	
C8	0.6999 (7)	0.29530 (15)	0.3773 (9)	0.0565 (14)	
H8A	0.6156	0.2991	0.4565	0.068*	
H8B	0.7590	0.2767	0.4400	0.068*	
C9	0.7821 (6)	0.33140 (15)	0.3632 (9)	0.0518 (13)	
H9A	0.8611	0.3280	0.2741	0.062*	
H9B	0.7198	0.3502	0.3088	0.062*	
C10	0.7249 (6)	0.35471 (16)	0.6839 (9)	0.0543 (14)	
H10A	0.6703	0.3326	0.7145	0.065*	
H10B	0.6608	0.3725	0.6241	0.065*	
C11	0.7848 (6)	0.37098 (15)	0.8671 (9)	0.0530 (14)	
H11A	0.7074	0.3775	0.9541	0.064*	
H11B	0.8448	0.3527	0.9312	0.064*	
C12	0.9850 (6)	0.39461 (16)	0.6932 (10)	0.0576 (15)	
H12A	1.0473	0.3766	0.7548	0.069*	
H12B	1.0407	0.4166	0.6633	0.069*	
C13	0.9238 (7)	0.37841 (16)	0.5096 (9)	0.0554 (14)	
H13A	0.8643	0.3969	0.4467	0.066*	
H13B	1.0012	0.3722	0.4221	0.066*	
C14	0.9037 (6)	0.42691 (14)	0.9829 (9)	0.0526 (14)	
C15	0.7973 (6)	0.44795 (15)	1.0736 (9)	0.0562 (14)	
C16	0.8248 (8)	0.47043 (17)	1.2283 (10)	0.0705 (18)	
H16	0.7505	0.4830	1.2892	0.085*	
C17	0.9637 (9)	0.47451 (18)	1.2945 (11)	0.078 (2)	
H17	0.9844	0.4903	1.3973	0.094*	
C18	1.0689 (8)	0.45489 (18)	1.2056 (11)	0.075 (2)	
H18	1.1626	0.4576	1.2482	0.090*	
C19	1.0410 (7)	0.43120 (16)	1.0548 (11)	0.0647 (17)	
H19	1.1155	0.4178	0.9997	0.078*	
C20	0.6040 (10)	0.4671 (2)	0.8848 (15)	0.085 (2)	

### Atomic displacement parameters (Å^2^)

**Table:**

	*U*^11^	*U*^22^	*U*^33^	*U*^12^	*U*^13^	*U*^23^
F1	0.088 (8)	0.111 (12)	0.117 (18)	0.016 (7)	−0.002 (10)	0.010 (13)
F2	0.113 (15)	0.109 (16)	0.091 (11)	0.022 (12)	−0.001 (9)	0.016 (10)
F3	0.100 (19)	0.091 (7)	0.122 (8)	0.022 (8)	0.005 (10)	0.012 (6)
F1'	0.09 (2)	0.11 (4)	0.12 (3)	0.02 (2)	0.00 (2)	0.01 (3)
F2'	0.09 (4)	0.103 (19)	0.113 (18)	0.03 (2)	0.00 (2)	0.005 (14)
F3'	0.10 (2)	0.105 (19)	0.11 (3)	0.014 (16)	−0.01 (2)	0.00 (2)
N1	0.071 (3)	0.069 (3)	0.046 (3)	−0.004 (3)	−0.001 (3)	0.000 (3)
N2	0.057 (2)	0.051 (2)	0.040 (2)	0.002 (2)	−0.001 (2)	0.005 (2)
N3	0.056 (3)	0.050 (3)	0.048 (3)	−0.002 (2)	0.003 (2)	0.001 (2)
O1	0.079 (3)	0.118 (4)	0.047 (3)	0.012 (3)	0.004 (2)	0.000 (3)
O2	0.066 (3)	0.058 (2)	0.075 (3)	0.008 (2)	0.007 (2)	0.006 (2)
O3	0.076 (3)	0.067 (2)	0.056 (3)	0.010 (2)	−0.013 (2)	0.005 (2)
O4	0.181 (6)	0.135 (5)	0.152 (7)	−0.048 (5)	0.062 (6)	−0.038 (6)
S1	0.0745 (11)	0.0845 (12)	0.0894 (14)	−0.0060 (9)	0.0120 (11)	−0.0241 (11)
C1	0.051 (3)	0.067 (4)	0.051 (4)	−0.014 (3)	0.003 (3)	−0.004 (3)
C2	0.051 (3)	0.063 (4)	0.061 (4)	−0.012 (3)	0.001 (3)	−0.001 (3)
C3	0.062 (4)	0.071 (4)	0.077 (5)	−0.003 (3)	−0.001 (4)	−0.003 (4)
C4	0.064 (4)	0.085 (5)	0.090 (6)	0.003 (3)	−0.006 (4)	0.015 (5)
C5	0.067 (4)	0.079 (5)	0.121 (8)	−0.001 (4)	−0.003 (5)	−0.004 (5)
C6	0.060 (3)	0.073 (4)	0.056 (4)	−0.004 (3)	−0.007 (3)	−0.005 (3)
C7	0.060 (3)	0.060 (3)	0.046 (3)	0.004 (3)	−0.006 (3)	−0.002 (3)
C8	0.059 (3)	0.064 (3)	0.046 (3)	0.000 (3)	−0.001 (3)	0.001 (3)
C9	0.058 (3)	0.058 (3)	0.039 (3)	0.002 (3)	−0.003 (3)	0.001 (3)
C10	0.055 (3)	0.056 (3)	0.052 (3)	−0.005 (3)	0.008 (3)	−0.001 (3)
C11	0.061 (3)	0.052 (3)	0.046 (3)	−0.003 (3)	0.009 (3)	0.005 (3)
C12	0.057 (3)	0.054 (3)	0.061 (4)	−0.002 (3)	0.007 (3)	0.002 (3)
C13	0.058 (3)	0.061 (3)	0.047 (3)	0.001 (3)	0.010 (3)	0.007 (3)
C14	0.067 (4)	0.043 (3)	0.048 (3)	0.000 (3)	−0.006 (3)	0.001 (3)
C15	0.068 (4)	0.048 (3)	0.053 (3)	0.004 (3)	0.000 (3)	0.000 (3)
C16	0.092 (5)	0.062 (4)	0.057 (4)	0.006 (3)	0.006 (4)	−0.008 (3)
C17	0.113 (6)	0.058 (4)	0.064 (5)	−0.003 (4)	−0.015 (4)	−0.012 (4)
C18	0.086 (5)	0.066 (4)	0.074 (5)	−0.010 (4)	−0.020 (4)	0.002 (4)
C19	0.075 (4)	0.051 (3)	0.069 (4)	−0.001 (3)	−0.009 (4)	−0.001 (3)
C20	0.076 (6)	0.087 (6)	0.092 (7)	0.015 (5)	−0.003 (5)	0.002 (5)

### Geometric parameters (Å, °)

**Table:**

F1—C20	1.331 (19)	C6—C7	1.526 (8)
F2—C20	1.33 (4)	C6—H6A	0.9700
F3—C20	1.35 (2)	C6—H6B	0.9700
F1'—C20	1.35 (8)	C7—C8	1.521 (9)
F2'—C20	1.34 (4)	C7—H7A	0.9700
F3'—C20	1.36 (4)	C7—H7B	0.9700
N1—C1	1.319 (8)	C8—C9	1.512 (8)
N1—C6	1.459 (8)	C8—H8A	0.9700
N1—H1	0.8600	C8—H8B	0.9700
N2—C13	1.459 (7)	C9—H9A	0.9700
N2—C10	1.463 (7)	C9—H9B	0.9700
N2—C9	1.468 (8)	C10—C11	1.504 (8)
N3—C14	1.415 (8)	C10—H10A	0.9700
N3—C12	1.447 (7)	C10—H10B	0.9700
N3—C11	1.465 (7)	C11—H11A	0.9700
O1—C1	1.247 (8)	C11—H11B	0.9700
O2—C20	1.300 (9)	C12—C13	1.509 (9)
O2—C15	1.418 (7)	C12—H12A	0.9700
O3—H21	0.8500	C12—H12B	0.9700
O3—H22	0.8500	C13—H13A	0.9700
O4—H23	0.8501	C13—H13B	0.9700
O4—H24	0.8500	C14—C19	1.386 (8)
S1—C5	1.694 (10)	C14—C15	1.400 (8)
S1—C2	1.709 (7)	C15—C16	1.365 (9)
C1—C2	1.480 (9)	C16—C17	1.386 (10)
C2—C3	1.351 (10)	C16—H16	0.9300
C3—C4	1.400 (10)	C17—C18	1.358 (10)
C3—H3	0.9300	C17—H17	0.9300
C4—C5	1.343 (13)	C18—C19	1.371 (10)
C4—H4	0.9300	C18—H18	0.9300
C5—H5	0.9300	C19—H19	0.9300
			
C1—N1—C6	123.0 (6)	N2—C10—H10A	109.4
C1—N1—H1	118.5	C11—C10—H10A	109.4
C6—N1—H1	118.5	N2—C10—H10B	109.4
C13—N2—C10	108.8 (4)	C11—C10—H10B	109.4
C13—N2—C9	108.5 (4)	H10A—C10—H10B	108.0
C10—N2—C9	111.9 (4)	N3—C11—C10	109.9 (5)
C14—N3—C12	116.7 (5)	N3—C11—H11A	109.7
C14—N3—C11	115.4 (5)	C10—C11—H11A	109.7
C12—N3—C11	109.8 (4)	N3—C11—H11B	109.7
C20—O2—C15	118.3 (6)	C10—C11—H11B	109.7
H21—O3—H22	104.0	H11A—C11—H11B	108.2
H23—O4—H24	107.0	N3—C12—C13	109.1 (5)
C5—S1—C2	91.7 (4)	N3—C12—H12A	109.9
O1—C1—N1	122.6 (7)	C13—C12—H12A	109.9
O1—C1—C2	119.8 (6)	N3—C12—H12B	109.9
N1—C1—C2	117.5 (6)	C13—C12—H12B	109.9
C3—C2—C1	130.3 (6)	H12A—C12—H12B	108.3
C3—C2—S1	111.4 (5)	N2—C13—C12	111.7 (5)
C1—C2—S1	118.2 (5)	N2—C13—H13A	109.3
C2—C3—C4	112.0 (7)	C12—C13—H13A	109.3
C2—C3—H3	124.0	N2—C13—H13B	109.3
C4—C3—H3	124.0	C12—C13—H13B	109.3
C5—C4—C3	113.6 (8)	H13A—C13—H13B	107.9
C5—C4—H4	123.2	C19—C14—C15	116.0 (5)
C3—C4—H4	123.2	C19—C14—N3	123.9 (5)
C4—C5—S1	111.3 (6)	C15—C14—N3	120.0 (5)
C4—C5—H5	124.3	C16—C15—C14	122.5 (6)
S1—C5—H5	124.3	C16—C15—O2	119.1 (6)
N1—C6—C7	112.4 (5)	C14—C15—O2	118.2 (5)
N1—C6—H6A	109.1	C15—C16—C17	119.9 (7)
C7—C6—H6A	109.1	C15—C16—H16	120.1
N1—C6—H6B	109.1	C17—C16—H16	120.1
C7—C6—H6B	109.1	C18—C17—C16	118.4 (7)
H6A—C6—H6B	107.9	C18—C17—H17	120.8
C8—C7—C6	113.6 (5)	C16—C17—H17	120.8
C8—C7—H7A	108.8	C17—C18—C19	121.9 (7)
C6—C7—H7A	108.8	C17—C18—H18	119.1
C8—C7—H7B	108.8	C19—C18—H18	119.1
C6—C7—H7B	108.8	C18—C19—C14	121.2 (6)
H7A—C7—H7B	107.7	C18—C19—H19	119.4
C9—C8—C7	111.8 (5)	C14—C19—H19	119.4
C9—C8—H8A	109.3	O2—C20—F2	114.8 (17)
C7—C8—H8A	109.3	O2—C20—F1	109.4 (10)
C9—C8—H8B	109.3	F2—C20—F1	110.2 (17)
C7—C8—H8B	109.3	O2—C20—F2'	113 (2)
H8A—C8—H8B	107.9	O2—C20—F1'	115 (3)
N2—C9—C8	114.7 (5)	F2'—C20—F1'	107 (4)
N2—C9—H9A	108.6	O2—C20—F3	114.3 (11)
C8—C9—H9A	108.6	F2—C20—F3	101.2 (14)
N2—C9—H9B	108.6	F1—C20—F3	106.3 (10)
C8—C9—H9B	108.6	O2—C20—F3'	109.3 (16)
H9A—C9—H9B	107.6	F2'—C20—F3'	109 (2)
N2—C10—C11	111.1 (4)	F1'—C20—F3'	103 (3)
			
C6—N1—C1—O1	1.4 (10)	C10—N2—C13—C12	57.5 (6)
C6—N1—C1—C2	−178.3 (5)	C9—N2—C13—C12	179.4 (5)
O1—C1—C2—C3	−177.8 (7)	N3—C12—C13—N2	−59.5 (6)
N1—C1—C2—C3	1.9 (10)	C12—N3—C14—C19	−19.7 (8)
O1—C1—C2—S1	0.1 (8)	C11—N3—C14—C19	111.5 (6)
N1—C1—C2—S1	179.8 (4)	C12—N3—C14—C15	157.5 (5)
C5—S1—C2—C3	1.7 (6)	C11—N3—C14—C15	−71.3 (6)
C5—S1—C2—C1	−176.6 (5)	C19—C14—C15—C16	−2.3 (9)
C1—C2—C3—C4	176.2 (6)	N3—C14—C15—C16	−179.7 (5)
S1—C2—C3—C4	−1.8 (8)	C19—C14—C15—O2	−177.7 (5)
C2—C3—C4—C5	1.0 (9)	N3—C14—C15—O2	4.9 (8)
C3—C4—C5—S1	0.3 (9)	C20—O2—C15—C16	88.0 (8)
C2—S1—C5—C4	−1.1 (6)	C20—O2—C15—C14	−96.5 (7)
C1—N1—C6—C7	−94.7 (7)	C14—C15—C16—C17	3.4 (10)
N1—C6—C7—C8	−64.5 (7)	O2—C15—C16—C17	178.8 (6)
C6—C7—C8—C9	−177.6 (5)	C15—C16—C17—C18	−1.9 (10)
C13—N2—C9—C8	175.9 (5)	C16—C17—C18—C19	−0.5 (11)
C10—N2—C9—C8	−64.1 (6)	C17—C18—C19—C14	1.6 (10)
C7—C8—C9—N2	−175.3 (5)	C15—C14—C19—C18	−0.2 (9)
C13—N2—C10—C11	−56.7 (6)	N3—C14—C19—C18	177.1 (6)
C9—N2—C10—C11	−176.5 (4)	C15—O2—C20—F2	60 (3)
C14—N3—C11—C10	166.0 (5)	C15—O2—C20—F1	−176 (3)
C12—N3—C11—C10	−59.6 (6)	C15—O2—C20—F2'	−89 (7)
N2—C10—C11—N3	58.4 (6)	C15—O2—C20—F1'	34 (6)
C14—N3—C12—C13	−166.8 (5)	C15—O2—C20—F3	−57 (3)
C11—N3—C12—C13	59.4 (6)	C15—O2—C20—F3'	150 (7)

### Hydrogen-bond geometry (Å, °)

**Table:**

*D*—H···*A*	*D*—H	H···*A*	*D*···*A*	*D*—H···*A*
N1—H1···O3^i^	0.86	2.17	2.929 (5)	148
O3—H21···N2^ii^	0.85	2.02	2.835 (4)	160
O3—H22···O1^iii^	0.85	1.98	2.822 (5)	171
O4—H23···O1^iv^	0.85	2.09	2.830 (7)	146
O4—H24···O3^v^	0.85	2.07	2.831 (6)	150

Symmetry codes: (i) *x*+1/2, −*y*+1/2, *z*; (ii) *x*−1, *y*, *z*; (iii) *x*−1/2, −*y*+1/2, *z*+1; (iv) *x*, *y*, *z*+1; (v) *x*+1, *y*, *z*.
